# Comparative study on skin protection activity of polyphenol-rich extract and polysaccharide-rich extract from *Sargassum vachellianum*

**DOI:** 10.1371/journal.pone.0227308

**Published:** 2020-01-07

**Authors:** Valentina Jesumani, Hong Du, Pengbing Pei, Muhammad Aslam, Nan Huang

**Affiliations:** 1 Guangdong Provincial Key Laboratory of Marine Biotechnology, College of Sciences, Shantou University, Shantou, Guangdong, China; 2 Faculty of Marine Sciences, Lasbela University, Uthal, Pakistan; Guangdong Technion Israel Institute of Technology, CHINA

## Abstract

Seaweed polyphenols and polysaccharide plays a broad range of biological activity. The objective of the present study was to study and compare the skin protection activity of fucoidan rich polysaccharide extract (SPS) and polyphenol-rich extract (SPP) from the seaweed *Sargassum vachellianum*. The skin protection activity was analyzed based on their ability to scavenge free radicals such as hydrogen peroxide and hydroxyl radicals, UV absorption potential, tyrosinase inhibition, moisture preservation, and antibacterial activity. From the results, both SPP and SPS protects the skin from UV damage. SPP showed good free radical scavenging ability, antimicrobial activity against *E*.*coli* and *S*. *aureus* and effectively absorbed the UVB and UVA rays whereas SPS hardly absorbs the UVA and UVB rays and showed weak free radical scavenging ability and no antimicrobial activity. SPS showed considerable inhibition on tyrosinase (51.21%) and had better moisture absorption (52.1%) and retention (63.24%) abilities than SPP. The results specified that both SPS and SPP have balancing potential on skin protection and suitable combinations of both could act as an active ingredient in cosmetics.

## Introduction

Skin is exposed to environmental stress like pollution and ultraviolet radiation from the sun. UV radiations are classified into UV-A (320 to 400nm), UV-B (290 to 320nm) and UV-C (100 to 290nm). UV-C is generally filtered by the earth's atmosphere, so the reduced amount of UVC radiations will extend to our skin. However, UVA and UVB rays reach the skin [1]. When the human skin is overexposed to UV radiation, it induces the Reactive Oxygen Species (ROS) production and subsequently generates oxidative stress. Consequently, these oxidative stress can lead to skin disorders such as hyperpigmentation (dark spots), premature aging and dryness [2,3]. ROS accumulation enhances the activity of tyrosinase which results in abnormal production of melanin, a pigment responsible for skin color. Tyrosinase is a copper-containing enzyme responsible for the physiological process of melanin synthesis caused by the hydroxylation of tyrosine [4]. In human skin, the overproduction and accumulation of melanin pigment result in hyperpigmentation. The inhibition of tyrosinase activity is a useful factor in calculating skin-lightening activity. In addition, Melanogenesis is described to result in the generation of free radicals like hydrogen peroxide in the melanocytes which again result in oxidative stress. Therefore ROS plays a significant role in the regulation of melanin synthesis and so ROS scavengers help in reduce the UV-induced melanogenesis [5]. Hyperpigmentation can be reduced by free radical scavengers and tyrosinase inhibitors that suppress melanogenesis in the skin [6]. Moisture absorption and retention capacity of the skin is getting disturbed due to age factor and exposure to environmental stress. Once the moisture in the skin is reduced, then the skin will become dry with wrinkles which also results in premature aging. Retaining moisture in the skin is also another important function of the skincare products. Compound that is rich in antioxidant activity associated with anti-tyrosinase and moisture-preserving properties and also has the ability to block the UV rays plays an important role in skin whitening and sunscreen products.

Many synthetic chemicals like arbutin, hydroquinone and kojic acid are being used as tyrosinase inhibitors in skin whitening product, but it was testified to side effect like dermatitis, genotoxicity and also induce cancer. Hence, the exploration of an effective skin care agent is still continuing in cosmetic research. In recent years, natural products have become more attractive in the cosmetic industry due to no side effects. The sea harbors a natural compound that is promising to act as biologically active metabolites. Marine algae are highly studied for their potent antioxidant capability [7,8,9,10]. The seaweed bioactive compound can play a vital role in skincare formulation because of its antioxidant properties. The active compound protects the skin in several ways, which includes scavenging the ROS, suppress the oxidation process, and protects the DNA damage. This antioxidant potential is mainly conferred by the presence of two important bioactive components such as polysaccharides and polyphenolic compounds [11]. Seaweeds exposed to extreme environmental stress including UV radiation induces the accumulation of polyphenols to defend from UV rays and act as a potent photo protector candidate. Earlier literature revealed that compounds such as phenolic compounds and polysaccharides are highly distributed in seaweeds which believed to be exhibiting higher antioxidative activities and also tyrosinase inhibition activity [12,13]. Fucose-rich sulfated polysaccharides called fucoidans present only in brown algae and echinoderms. These polysaccharides diverge in their structure, composition and sulfation arrangements based on the species, and extraction methods.

*Sargassum* is the largest genus in the Phaeophyta in which more than 130 species can be found in China. *S*. *vachellianum* is ecologically significant brown algae that reside in the subtidal zone of the coasts of China. Its bioactivities are not still explored. In order to harness the potentiality of the bioactive compounds of *S*. *vachellianum* in the field of cosmetics, we have extracted polyphenol-rich (SPP) and fucoidan rich polysaccharide (SPS) extracts. Though it is evident that both polysaccharide and polyphenol are potential in antioxidant activity, the comparison of the phenol-rich fraction (SPP) and a polysaccharide-rich fraction (SPS) of seaweed has not been studied with regards to skin-protecting effect. So it is noteworthy to study and compare the potential of both SPP and SPS as a skincare agent in detail. In the present study, the skin protection ability of the polyphenol (SPP) and polysaccharide (SPS) rich extracts of most unexplored seaweed *S*. *vachellianum* was studied on the following aspects: free radical scavenging activity, UV absorption potential, moisture absorption, and retention ability, tyrosinase inhibition activity and antibacterial activity.

## Materials and methods

### Materials

*Sargassum vachellianum* was collected from Nanao’ island, Guangdong Province in China s (23° 17′ 50” N and 117° 04’ 46” N) during March 2018. The bacterial strains, *Escherichia coli and Staphylococcus aureus* were procured from the lab of Resources and Environmental Microbiology, Shantou University, Shantou, China. Folin Ciocalteau, DPPH, L- DOPA, tyrosinase, kojic acid were purchased from Sigma-Aldrich. Analytical grade organic solvents were used for the sample preparation.

### Extraction of Polyphenol-rich fraction (SPP) and Polysaccharide rich fraction (SPS)

The collected seaweed *S*. *vachellianum* was processed to remove the attached specimens on its surface. Then, the seaweed was washed carefully in tap water and then in distilled water. The processed seaweeds were dried under shade, powdered and stored in airtight containers until used for extraction.

**Fig 1:**
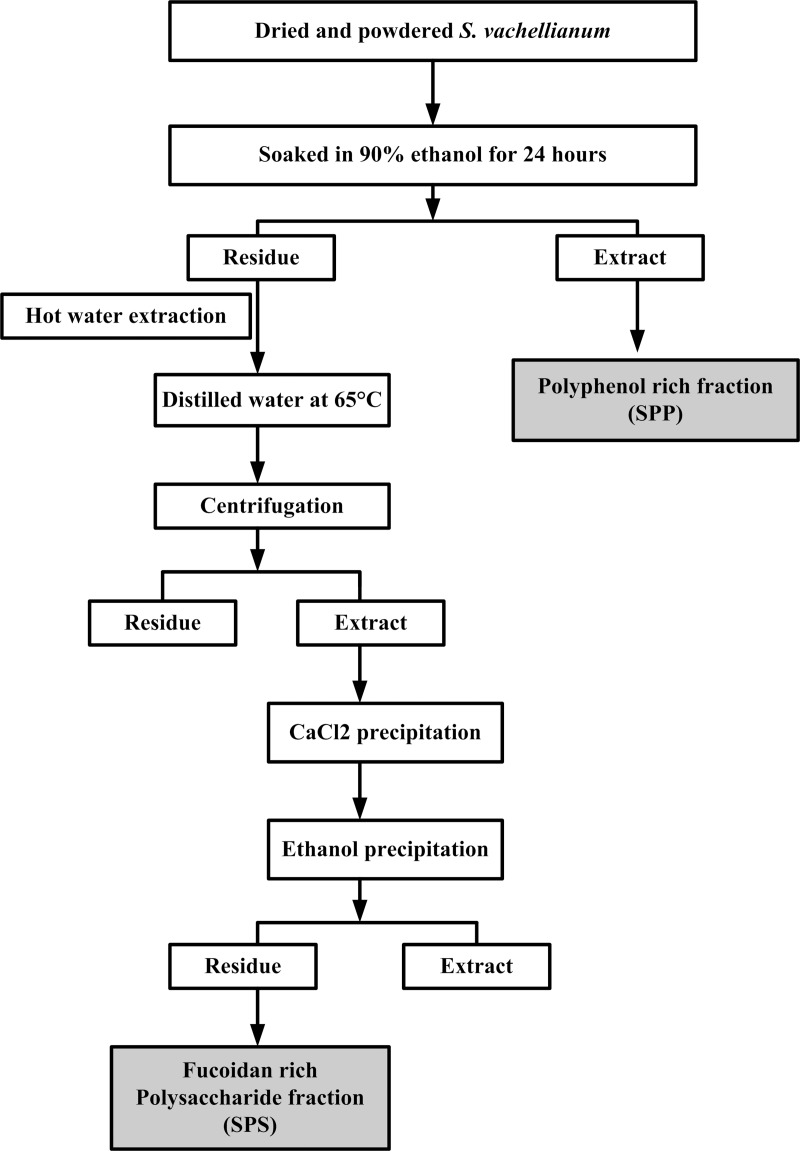
Overview of the process to prepare polyphenol-rich fraction (SPP) and a polysaccharide-rich fraction (SPS) from *S*. *vachellianum*.

The powdered seaweed (30g) was soaked in 300mL of 90% ethanol and kept in a shaker for 24h. After centrifugation, the residue was collected. The soluble solvent was filtered and the same extraction procedure was repeated 2 times. The filtered portion from the extraction was pooled together and the solvent was allowed to evaporate to dryness at 40°C using a rotary evaporator under reduced pressure. Once drying, the extracts were weighed up and stored until used. The sample is dissolved in ethanol to provide the crude ethanolic extract rich in polyphenols (SPP 8.12%). The residue from the ethanol extraction was mixed with 200 ml of distilled water at 65°C under stirred condition for 1h. Then centrifuged the extracts at 15,000 rpm for 15 min and the supernatant was collected and mixed with CaCl_2_ and kept at 4°C overnight to eliminate the alginate. Three volume of 99% ethanol was added to the supernatant in order to precipitate the polysaccharide and kept at 4°C overnight. The precipitate was then freeze-dried to extract the crude fucoidan rich polysaccharide fraction (SPS 5.5%) (Fig 1).

### Chemical composition

Total phenolic content was estimated by using the Folin-Ciocalteu assay [10]. In brief, 0.75 mL of folin-ciocalteau reagent (1:9; folin-ciocalteau: H_2_O) and 0.1 mL of sample (1 mg/ ml) were put in a test tube. Incubated the mixture at room temperature for 5 minutes and 0.75 mL of 6% (W/V) Na_2_Co_3_ was added. The mixture was mixed well and incubated at room temperature for 90 minutes. The absorbance was measured at 765 nm using a UV-Vis spectrophotometer. The total sugar was calculated by the phenol-sulfuric acid method [14]. Briefly, 200 μL of SPS, 200 μL of 5%, w/v phenol solution and 1.0 mL con sulfuric acid was added and incubated for 20 min at room temperature. The absorbance was measured at 490 nm. Glucose was used as a standard. Sulfate content was determined by the barium sulfate method [15]. After acid hydrolysis of the sample with 200 μL of 1 M HCl for 2 h at 100°C, the volume of 3.8 mL of 3%trichloroacetic acids and 1.0 mL of a barium chloride-gelatin solution was added and incubated for 15 min. The sulfate content was calculated by the absorbance at 360 nm using K_2_SO_4_ as a standard. Monosaccharide composition of the TFA hydrolyzed sample was analyzed by HPLC using 1-phenyl-3-methyl-5-pyrazolone derivative sample according to the method of Cheong et al. [16].

### Fourier transform infrared spectroscopy analysis

Fourier transforms infrared (FTIR) spectroscopy was used to analyze and find the functional group of polysaccharides and polyphenols. 1 mg of powder of polyphenol extract and polysaccharide extract was mixed with anhydrous potassium bromide and the spectra were recorded from 500 to 4000 cm-1.

### Skin protection activities

#### Hydrogen peroxide radical scavenging assay

H2O2 scavenging activity of the sample was determined based on Ruch et al. [17]. 2 ml of 40mM H2O2 solution prepared in 0.1 M phosphate buffer pH 7.4 was added to 1ml of various concentrations of the sample and incubated for 10 min at room temperature. The absorbance was measured at 230 nm using phosphate buffer as a blank solution. Ascorbic acid was used as a standard. The percentage of scavenging activity was calculated as

$$scavenging\;activity\;(\%)=\left(1-\frac{A\;sample}{A\;control}\right)\times 100$$

#### Hydroxyl radical scavenging assay

The HO scavenging ability of the samples was calculated by a Fenton-type reaction [18]. 1ml of different concentrations of the sample was mixed with 1 ml of 9mM ferric sulfate, 1ml of 9mM ethanolic salicylic acid and 1ml of 9mM hydrogen peroxide. The mixture was incubated for 30min at 37°C. The absorbance was measured at 510 nm. Ascorbic acid was used as a positive control. The activity was calculated as above.

#### Determination of UV absorption potential

For UV absorption potential activity, the sample SPP and SPS were mixed with a suitable solvent at the concentration of 1000 μg/mL. The solvent was used as a blank. Samples were observed for absorbance at 200–700 nm wavelength (particularly at the critical wavelength of 290 and 310 nm for UVB range, 330 and 350 nm for UVA range were chosen for comparison) using dual-beam UV Spectrophotometer [19].

#### Anti tyrosinase activity

The inhibitory effect of SPP and SPS on tyrosinase activity was measured spectrophotometrically described by Chan et al [20]. The activity was calculated based on the amount of inhibition on oxidation of L–DOPA by tyrosinase. The mixture of mushroom tyrosinase (700 units/ml) prepared in 0.1 M of phosphate buffer pH 6.8 and 0.5 mM of L-DOPA in 0.1 M phosphate buffer. 50μl of the enzyme was added in the 96-well plate containing 50 μl of different concentrations of SPP /SPS and 100μl of phosphate buffer. The samples were incubated at room temperature for 10 min in which 100 μl of the substrate was added and incubated at room temperature for 20 min. The amount of dopachrome formed was measured at 475 nm in a microplate reader. Kojic acid was used as a standard and phosphate buffer was used as blank. The percentage of inhibition was calculated as above.

#### Moisture absorption and retention test

Moisture absorption and retention ability were tested based on the method explained by Shao et al. [21]. The samples were oven-dried for 4 hours and make sure the samples were dried completely. A known amount of the sample was taken in the vial and the initial weight of the sample was weighed. Then the sample was kept in the saturated (NH_4_)_2_SO_4_ desiccator which is 81% relative humidity. The ability of water absorption was calculated based on their increase in weight. The observation was made in every 12 hours and expressed the ability by the percentage of increase in weight of the dry sample:

$$Moisture\;absorption\;(\%)=(\frac{Wn-W0}{W0})\times 100$$

In the moisture-retention test, the known amount of dried sample was mixed with the known amount of water (10%). The weight of the wet sample was weighed and then kept in the saturated K_2_CO_3_ desiccator which is 43% relative humidity. The moisture-retention ability was observed at every 12 hours and calculated by the percentage of the remaining water in the wet sample.

$$Moisture\;retention\;(\%)=\frac{Wn}{W0}\times 100$$

Where W0 and Wn were the initial and final weights of the sample.

#### Antibacterial activity

The antibacterial activity of SPS and SPP against Gram-positive and Gram-negative bacteria was determined by the disk diffusion assay. Bacteria used such as *Escherichia coli and Staphylococcus aureus* were maintained in nutrient broth. Nutrient agar plates were prepared and 100 μL of the bacterial culture (10^6^ CFU) was spread. The paper disc containing a sample (1mg/mL) was placed over the agar plates and incubated for 24 hours at 37°C. Ampicillin (10 μg/mL) was used as a positive control After incubation, the diameter of the inhibition zone was measured.

#### Minimum Inhibitory Concentration (MIC)

Minimum Inhibitory Concentration was calculated by the two-fold dilution method. MIC was referred to as the minimum concentration of sample which inhibited the growth of bacteria at 37°C. Different concentrations of the sample were prepared (0.2, 0.4, 0.6, 0.8, 1mg/mL) by two-fold dilutions. The culture was inoculated and incubated for 24 hours at 37°C. After incubation the minimum concentration that shows no visible growth was noted.

### Ethical statement

This study did not involve any protected species or animals. So any specific permit was not required for the present study.

### Statistical analysis

Statistical analysis was done by the SPSS software package. The results of the experiments were indicated as mean ±SD of triplicates adopted.

## Results

### Composition of the extracts

The extraction yield was recorded as 8.12±0.35% and 5.5±0.25% for SPP and SPS respectively. The SPS showed the maximum presence of sugar (53.51%), sulfate (12.32%) and low polyphenol content (6.21%). The monosaccharide composition also showed the maximum presence of fucose followed by mannose. Conversely, the SPP showed the maximum presence of polyphenol (38.62%) and low sugar content (2.94%) with a minor quantity of fucose and galactose (Table 1).

**Table 1:** The chemical composition of SPP and SPS.

Seaweed composition	SPS	SPP
Yield (%)	5.5±0.25	8.12±0.35
Total Sugar (%)	53.51±0.54	2.94±0.71
Sulfate (%)	12.32±0.33	1.05±0.21
Protein Content (%)	1.52±0.46	1.13±0.81
Total phenolic content (%)	6.21±0.14	38.62±0.34
Fucose (%)	49.5	ND
Glucose (%)	2.2	1.96
Galactose (%)	9.3	1.05
Xylose (%)	3.5	ND
Mannose (%)	11.2	ND
Glucuronic acid (%)	1.01	ND

Each value is expressed as mean ± SD (n = 3)

### Characterization of SPP and SPS by FT-IR analysis

**Fig 2:**
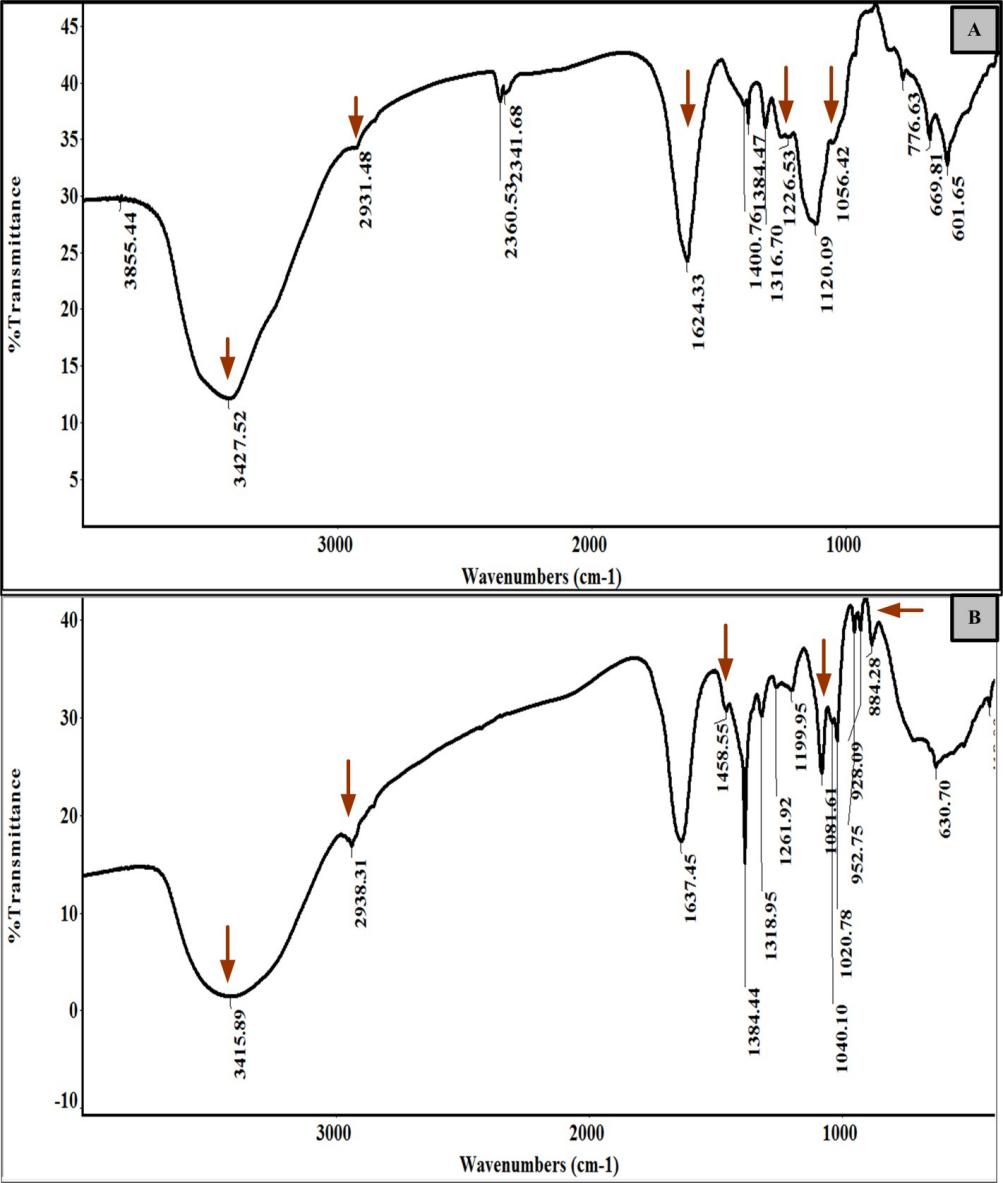
FT-IR spectra of extract. (A) SPS and (B) SPP.

Fig 2 showed the FTIR spectrum of SPP and SPS which exhibited the characteristic band features. SPS showed the broadband positioned at 3487, asymmetric vibration at 1624 and weak signals at 2937, 1226 and 1056 represents the presence of sulfated polysaccharides. Whereas, the broad peaks at 3415 and weak signals at 2938 and 1458 represents the presence of phenols in SPP. The characteristic peak at 1618 cm-1 and stretched vibration at 1384 represents the combined C-O stretching vibration with stretched C-C ring of phenyl. The vibrations at 1081, 884 cm−1 confirmed the presence of aromatic polyphenols in SPP.

### Skin protection activity

#### Radical scavenging activity of SPP and SPS

**Fig 3:**
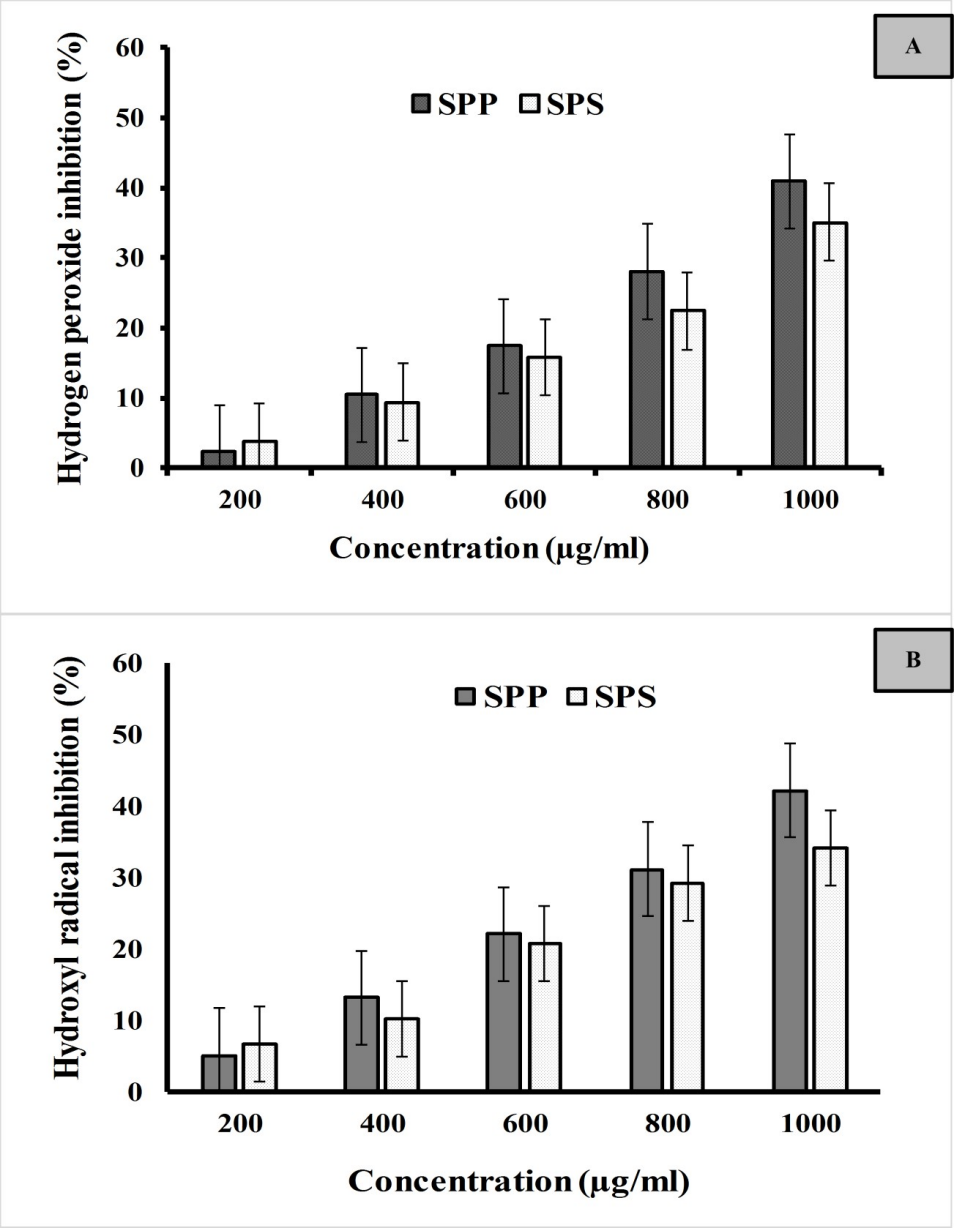
Radical scavenging activity of SPP and SPS. (A) Hydrogen peroxide (B) Hydroxyl radical scavenging activity.

Hydroxyl radical and hydrogen peroxide radical scavenging activity of SPS and SPP are shown in Fig 3. The SPP and SPS showed the scavenging activity of–OH in a concentration-dependent way. The inhibition percentage at the concentration of 1000μg/ml was 42.21± 0.32% for SPP and 34.12± 0.42% for SPS. The maximum scavenging activity of hydrogen peroxide radical was recorded by the SPP with the inhibition percentage of 40.88 ± 0.15%, whereas the SPS exhibited 35.12 ± 0.41% at the concentration of 1000μg/ml.

#### UV-sunscreen potential

**Fig 4:**
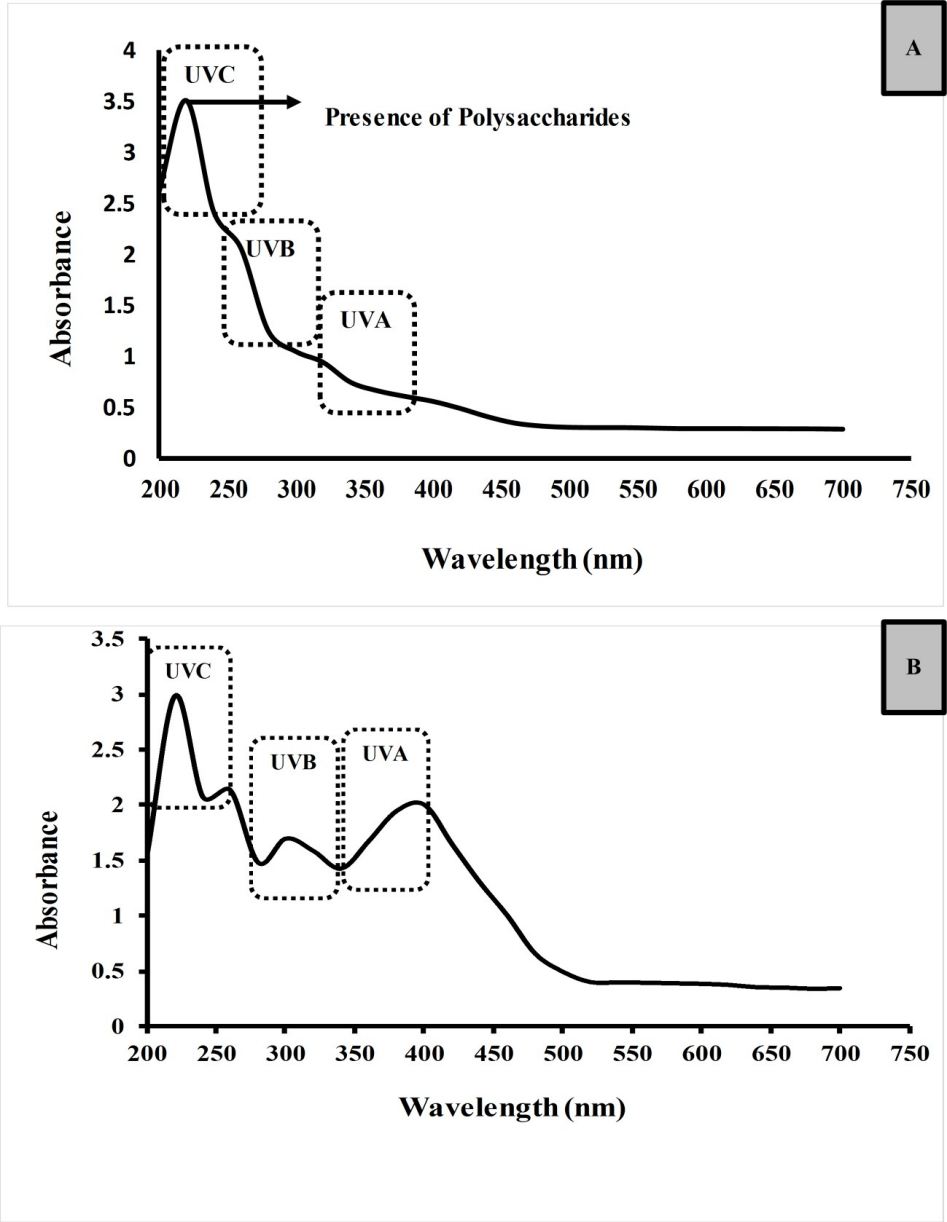
UV absorption spectra. (A) SPS and (B) SPP.

UV absorption activity of the SPP and SPS based on the absorbance of the sample at the spectrum of 290 – 315nm which is UVB rays and 315 – 400nm for UVA rays are presented in Fig 4.

#### Anti tyrosinase activity

**Fig 5:**
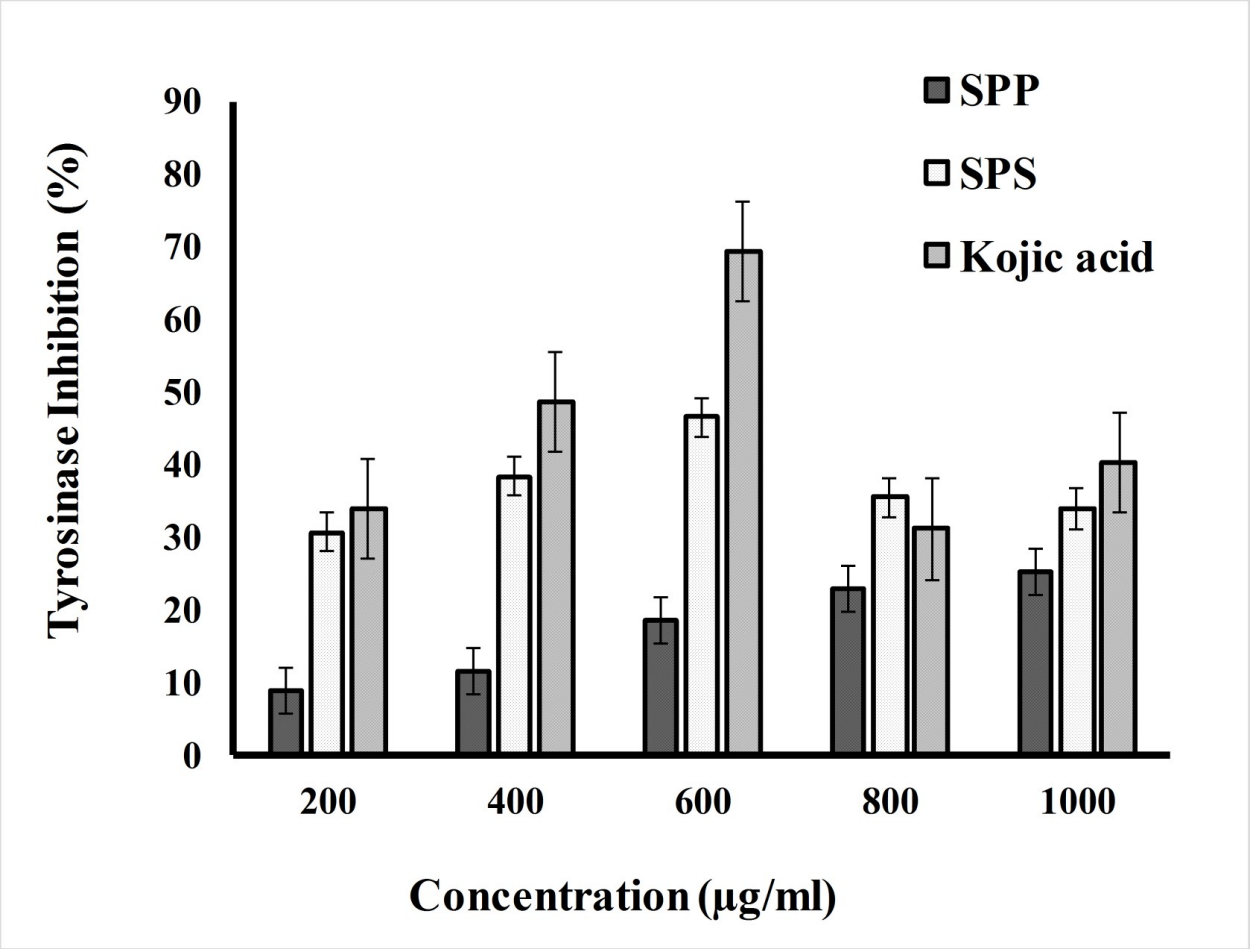
Tyrosinase inhibition activity of SPS and SPP.

The tyrosinase inhibitory activity of SPP, SPS are shown in Fig 5. Within the range of 200 to 1000 μg/ml, the inhibition percentage varies between 9.09 to 35.4% for SPP and 25.12 to 51.21% for SPS. This demonstrated that the inhibition ability of SPS showed well at the lower concentration when compared to SPP, whereas the kojic acid showed the inhibition percentage ranged between 11.01 to 40.21% in the concentration range of 20 to 100 μg/ml.

#### Moisture absorption and retention efficiency test

**Fig 6:**
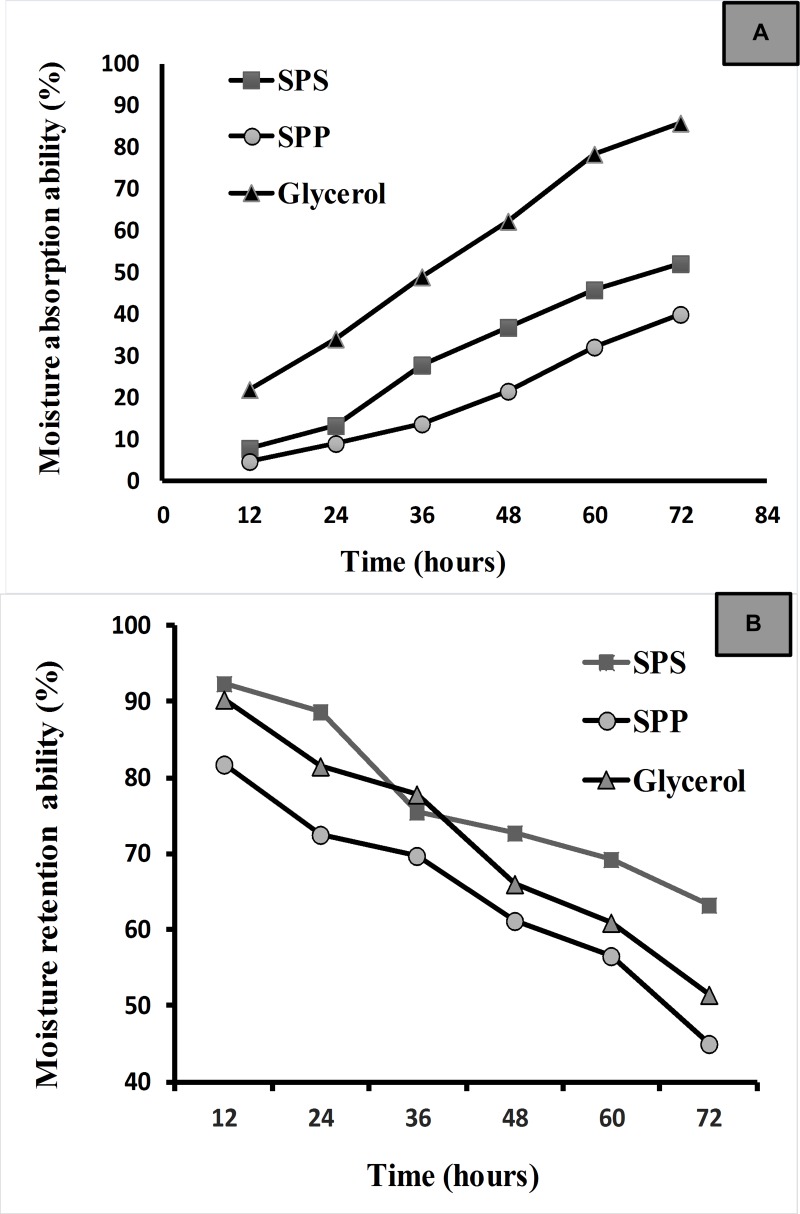
The moisture absorption and retention ability of SPP and SPS. (a) Moisture absorption (RH = 81%) (b) retention ability (RH = 43%).

The Moisture Absorption and Retention ability of SPP and SPS are presented in Fig 6. The absorption rate on 72h at 80% RH for SPS and SPP was recorded as 50.5% and 40% respectively. After 72h, the moisture retained in the SPS was 65.84% which was higher than SPP (45%) and glycerol (51.35%).

#### Antibacterial activity

The antibacterial activity of SPS and SPP is summarized in Table 2 The SPS did not show any inhibition against the tested bacteria whereas SPP showed maximum inhibition against *S*. *aureus* with 12.3mm and 7.2mm of inhibition zone for *E*.*coli*. The standard showed 24.6mm and 21.0mm of inhibition zone for *S*. *aureus* and *E*.*coli* respectively.

**Table 2:** Antibacterial activity of SPS and SPP.

Sample	*S*. *aureus*		*E*. *coli*	
	Zone of Inhibition (mm)	MIC (mg/mL)	Zone of Inhibition(mm)	MIC (mg/mL)
SPP	12.3±1.21	0.4	7.2±0.66	0.8
SPS	NA	NA	NA	NA
**Ampicillin**	24.6±0.09	0.009	21.0±0.33	0.03

NA- No activity: Zone of Inhibition including the diameter of 6 mm paper disc: MIC–Minimum inhibition concentration.

## Discussion

### Chemical composition and Characterization of SPS and SPP

The present study aimed to compare the skin protection activity of seaweed polysaccharide and polyphenol extract. The dried seaweed was extracted with ethanol to obtain the polyphenol-rich extract (SPP) and the water-soluble polysaccharide (SPS) rich extract was obtained by hot water extraction and calcium and ethanol precipitation. From Table 1, it is clear that SPP composed of polyphenols whereas SPS contains mainly carbohydrates with fucose as a major sugar. Charoensiddhi et al. [22] reported the maximum presence of sugar in water-soluble fraction and maximum phenol content in the ethanol-soluble fraction. Barros-Gomes et al. [23] also showed the maximum presence of the phenolic compound in methanol extract and maximum sugar content in the aqueous extract. These studies support our results. Generally, alcohol is used to precipitate the polysaccharides, polysaccharides have many hydroxyl groups which easily forms hydrogen bonds with water than with alcohol [24]. It was shown that the hot water extraction of *S*. *vachellianum* yielded sulfated polysaccharide with the maximum presence of carbohydrate which was estimated as 53.51±0.54% and sulfate content of 12.32±0.33% which in agreement with previous studies with brown seaweeds [25]. The sulfate group present in the SPS has been found to be important for bioactivity [26]. It was also showed the minimum presence of phenol 6.21±0.14%. When extracted in the low temperature may also result in the extraction of traces of phenolic compound, which attach to the hydroxyl group of monosaccharide molecules in the polysaccharide chain [27]. The composition of SPP showed that the fraction is rich in fucose-containing sulfated polysaccharide generally called fucoidan. Phlorotannin is an important phenolic compound present in brown seaweeds [28]. Matanjun et al [29] stated that the brown seaweeds have maximum phenolic content than in red and green seaweeds which in agreement with the present study.

Major functional groups namely OH, CH and CO were identified by evaluating the FTIR spectrum. Fig 2A showed the FTIR spectrum of SPS which exhibited the characteristic band features. The band at 3427cm -1 and 2931cm -1 signifies the presence of O-H and C-H groups correspondingly which determined the extracted samples are polysaccharides. The stretching at 1,226 cm−1 identifies the S = O vibration showed the presence of the sulfate group. The stretching at 1056 cm−1 represented the vibration related to the C-O-S = O group, endorsing the presence of sulfated polysaccharides in SPS. The deep band at 1624 cm−1 could be recognized as the vibration of C = O represents the uronic acid residues. These peaks in SPS confirmed the presence of fucoidan polysaccharides in the extract. Fig 2B showed the FTIR spectrum of SPP. The band at 3415 and 2938 cm ^-1^ found in SPP represents the stretching vibrations of phenolic O-H and C-H groups respectively that indicated the presence of alcohols and phenols. The stretching at 1637 specifies the presence of C = C cyclic. The peak at 1458 cm−1 denoted the bending vibrations related to CH_2_-CH_3_. The intense band at 1081–952 cm−1 could be due to vibrations of the C-O stretch [30]. The bands detected at 884 showed C-H aromatics confirmed the occurrence of polyphenols. These peaks in SPP confirmed the presence of polyphenol [31,32,33].

### Skin protection ability

It has been described that free radicals generated by ultraviolet light induce oxidative stress and accelerate skin related problems like hyperpigmentation and skin aging [34]. The determination of the antioxidant activity is the preliminary step in the assessment of skin protection activity. Antioxidant activity of SPP and SPS were evaluated based on the scavenging activity of the free radicals such as hydrogen peroxide and hydroxyl radicals. Hydroxyl is the most dynamic free radical that crosses the cell membranes easily react with the biomolecules like DNA, lipids, and protein which leads to cell damage and ultimately results in cell death. As shown in Fig 3. The SPP and SPS showed the scavenging activity of–OH in a concentration-dependent way. The IC50 value of SPP, SPS and ascorbic acid were 0.98, 1.31 and 0.12 mg/ml respectively. The results proved that the SPP is more potential in scavenging the–OH radical than SPS. The antioxidant mechanism for scavenging the hydroxyl radical involves that antioxidants may join to the metal ions and react with H_2_O_2_ which forms the metal complexes. These metal complexes hinder further reaction with H_2_O_2_ to give hydroxyl radicals [35]. In previous literature, it was reported that the scavenging activity of algal polysaccharide against hydroxyl radical seems to be moderate [36]. This proved that the maximum scavenging ability of SPP against hydroxyl radicals was due to the presence of high phenol content. Hydrogen peroxide itself is not reactive, but it produced the dangerous free radical like hydroxyl radical (•OH) when hydrogen peroxide reacts with Fe (II) and Cu (II) which results in cellular toxicity. Therefore, the elimination of H_2_O_2_ is very essential for antioxidant defense in the cell. Hydrogen peroxide scavenging capacity of SPP and SPS was studied. Both the seaweed extract SPP and SPS exhibited considerable scavenging activity. The inhibition increased with the increase in concentrations. The IC50 value of SPP, SPS and ascorbic acid were 0.8, 1.12 and 0.09 mg/ml respectively.

Many studies demonstrated the significant correlation between phenolic content and scavenging activity. The phenolic compounds produced as an antioxidant defense by the seaweed when exposed to environmental stress. Previous literature proved the potent antioxidant activity of brown. Studies on different Sargassum species: *Sargassum marginatum*, *S*. *wightii*, *S*. *fusiforme*, *S*. *fulvellum*, *S*.*siliquastrum*, and *S*. *swartzii* showed the potential free radical scavenging activity. These studies also suggested that the extract with more phenol content result in a high antioxidant activity which in agreement with the present study [36,37,38,39]. It was stated that the hydroxyl group in the polysaccharides and aromatic ring present in the phenol groups are responsible for the antioxidant activity [39]. The existence of low activity in the scavenging of hydrogen peroxide and hydroxyl radicals is common in brown algae polysaccharides. This indicates that the removal of hydroxyl radicals may not be the key antioxidant mechanism of polysaccharides [25].

One of the ways in which the sunscreen products protect the skin is by absorbing the UV radiation before reaching the skin. When the human skin is overexposed to UV radiation at the wavelength of 320–400 nm which is designated as UVA and UVB with a wavelength of 290 to 320 nm reaches the skin which leads to suntan, wrinkles, and hyperpigmentation. So the present study aims to show the absorption ability of the SPP and SPS at the critical wavelength corresponds to UVA and UVB. In Fig 4A, The SPP showed the peak at 270nm (UV- C range 100- 290nm), 312nm (UV- B range 290–315nm) and 396nm (UVA range 315–400nm). UV absorption improved exponentially from 350 nm to 400 nm, which relates to the UVA range. Whereas SPS showed weak absorption of UV-A and UV-B when compared to SPP. The variations in the UV absorption depends on the compound present in the sample. It is likely to compare the antioxidant potential of the seaweeds based on the bioactive compounds analyzed by UV-vis absorption spectrum. SPS showed a peak between 200 to 270nm which corresponds to polysaccharide bound covalently with aromatic compounds. Same kind of result observed with the fucoidan from *Saccharinna japonica* [40]. Whereas, the absorption peak of the SPP between 266 and 374nm determines the presence of phenolic compounds [32]. The Phenolic compounds received more attention for their potential roles in UV photoprotection and ROS scavenging [41]. Therefore, SPP showed good potential in absorbing UVA and UVB rays and scavenging ROS which might be due to the presence of benzene ring in polyphenols [11]. The present result is in good agreement with the report of Fitton et al [42] in which polyphenol-rich fraction of *Fucus vesiculosus* showed good UV absorption than the fucoidan rich fraction of *Undaria pinnatifida*. The SPP which showed the better potential in scavenging the free radicals and absorbing the UV rays could consider being used in the sunscreen formulation which protects the skin by preventing the penetration of UV rays and the damage caused by the UV rays.

Melanin is the pigment responsible for the skin and could be overproduced sometimes due to chronic exposure to the sun. Tyrosinase is a copper-containing enzyme that catalyzes the melanin synthesis. Thus the production of melanin can be controlled by inhibiting the tyrosinase activity. The ability of SPP and SPS to inhibit tyrosinase activity can be explained their perspective as a skin whitening agent. The tyrosinase inhibitory effect of SPP and SPS using L-DOPA was studied using the enzyme mushroom tyrosinase which is responsible for the formation of L- Dopa. Kojic acid and SPP showed an increase in inhibition in a dose-dependent way, whereas SPS showed an increase in inhibition up to the concentration of 800 μg/ml with 51.21± 0.46% inhibition percentage. The results suggested that SPP and SPS both exhibited positive tyrosinase inhibition activity but SPP showed weak activity than SPS. This may be due to the interference of some components present in the crude extracts. Moreover the difference in the activity also primarily based on the extraction parameters and also the structure of the compound. Fernando et al. [43] studied the tyrosinase inhibition of fucoidan from *Sargassum polycystum* which showed 20% of inhibition at the concentration of 200 μg/ml which is lesser than the present result. Whereas the ethanol crude extract of *S*. *polycystum* did not demonstrate any effect on mushroom tyrosinase inhibition assay which supports the present findings [20]. Anti tyrosinase properties may depend on species, habitats, harvesting time and condition. Based on these findings the SPS could be incorporated in the skin whitening formulation due to its ability to tyrosinase inhibition along with free radical scavenging activity.

The moisture content in the skin usually determines the skin health, hence maintaining the moisture is one of the key factors in skin care products. The moisture absorption and retention properties of SPP and SPS were studied in vitro for 72h and compared with glycerol, which is commonly used as a humectant and hygroscopic agent. As shown in Fig 6, the absorption ability of moisture in SPP increased slowly in the beginning until 48h at 80% relative humidity. Then slowly it started to increase in weight which means the increase in absorption at 72 hours. Whereas SPS showed better moisture absorption ability when compared to SPP. The absorption was slow till 36h after that the weight of the sample continued to increase till 72hours at 80% relative humidity. The absorption rate on 72h at 80% RH for SPP and SPS was recorded as 50.5% and 40% respectively.

The moisture retention ability of SPS and SPP were studied in saturated K_2_CO_3_ desiccator for 72h. The moisture retention capacity was calculated based on the percentage of water loss in the sample and the results were shown in Fig 6. Both SPP and SPS lost the moisture slowly with an increase in time. But the water loss in SPS was slower than SPP and glycerol, which means SPS could able to retain the water. Both moisture absorption and retention ability of SPS were higher than SPP which concluded that polysaccharide-rich extract (SPS) has the strongest moisture preserving ability which can be used as a moisturizer. The same result was absorbed by Wei et al. [11], where the green tea polysaccharide exhibited better moisture absorption and retention ability than green tea polyphenol which in line with the present study. The higher moisture absorption and retention ability of the polysaccharide-rich sample are due to the binding of polar groups, hydroxyl and carboxyl groups of the polysaccharide with water molecules through the hydrogen bond which helps the polysaccharide to absorb and hold the water. At the same time, the molecules in the polysaccharide could also jointly link together to form a frame-like structure, which allows the water to retain in the polysaccharide. Thus the SPS has strong moisture absorption and retention ability [44]. Generally, polysaccharides from brown seaweeds displayed the best moisture-absorption and retention ability compared to polysaccharides from red or green algae [26].

The antibacterial activity of the cosmetic ingredients helps in maintaining the skin microflora and also used as a preservative. The *S*. *aureus* is the normal flora present in the human skin surfaces. These are inoffensive in normal conditions but when the skin is wounded, these bacteria can pass the wound and cause infections. The secretion of bacterial toxins on the skin epidermis can cause pimples and blisters [13]. Atopic dermatitis is a skin disease commonly inhabited by *S*.*aureus* later intensifies the disease by inducing further inflammations [45]. Microorganisms like *S*. *aureus* and *E*.*coli* are also responsible for skin and soft tissue infections [46]. The antibacterial activity of SPP and SPS was analyzed against gram-positive *S*. *aureus* and gram-negative *E*.*coli*. The SPS did not show any antibacterial activity, whereas SPP showed the maximum activity on *S*. *aureus* than *E*.*coli*, but lesser than ampicillin. Previous studies reported that antimicrobial activity depends on the chemical structure and phenol content which also supports the present outcomes. The phenol-rich fraction exhibited better antimicrobial activity than the polysaccharide-rich fraction. Alboofetileh et al. [47] proved the antimicrobial activity of the fucoidan extracted by a different method and showed the fucoidan extracted by hot water did not show any activity which agreed with our present investigation. The antimicrobial activity also depends on the cell wall of the bacteria. The SPP showed better activity in gram-positive bacteria, as it has thick peptidoglycan but no outer membrane which allows the compound to penetrate and inhibit the growth.

## Conclusion

The results of the present work suggested that both fucoidan rich polysaccharide extract (SPS) and polyphenol-rich extract (SPP) has good protection on skin based on their antioxidant ability, protection against bacteria, whitening effect, moisture-retaining ability and also UV rays absorbing ability. Among which SPP showed good scavenging ability against hydrogen peroxide and hydroxyl radicals than SPS. Whereas SPS showed better tyrosinase inhibition and moisture absorption and retention ability than the SPP. SPP could strongly absorb the UVA and UV B whereas SPS absorbed the UV rays but less than SPP. These results provided useful data for formulating skin care products. The results also suggested that the combination of SPP and SPS would be a promising ingredient for skin protection. The present observation is based on the *in vitro* studies. Further, *in vivo* studies and investigations on the active compound and underlying mechanism involved in the skin protection activity are need to be studied in the future.
